# Methyl *N*-methylanthranilate: major compound in the defensive secretion of *Typhloiulus orpheus* (Diplopoda, Julida)

**DOI:** 10.1007/s00049-017-0242-4

**Published:** 2017-07-24

**Authors:** Michaela Bodner, Boyan Vagalinski, Slobodan E. Makarov, Günther Raspotnig

**Affiliations:** 1 Institute of Zoology, University of Graz, Universitätsplatz 2, 8010 Graz, Austria; 2 Institute of Biodiversity and Ecosystem Research, Bulgarian Academy of Sciences, 2 Gagarin Street, 1113 Sofia, Bulgaria; 3 Faculty of Biology, Institute of Zoology, University of Belgrade, Studentski Trg 16, 11000 Belgrade, Serbia

**Keywords:** Chemical defense, Quinone millipedes, Julidae, Methyl *N*-methylanthranilate

## Abstract

The defensive secretion of the julid diplopod *Typhloiulus orpheus* contains methyl *N*-methylanthranilate (MNMA), an ester of *N*-methylanthranilic acid that comprises more than 99% of secretion of this species. MNMA is accompanied by small amounts of methyl anthranilate and two benzoquinones (2-methyl-1,4-benzoquinone and 2-ethyl-1,4-benzoquinone, respectively). MNMA is a known intermediate in the biosynthesis of both benzoquinones (as present in defensive secretions of juliformians) and glomerin-like quinazolines (chemical defense in Glomerida). The compound may have evolved independently in the pathway to glomeridan chemistry, or may even represent a pivotal branching point in the pathway to different chemical classes of diplopod defensive chemistry.

## Introduction

Juliformian diplopods have traditionally been considered to possess uniformly composed defensive secretions, mainly comprising an array of substituted benzoquinones and hydroquinones (“quinone millipedes” sensu Eisner et al. 1978). More than 90 juliformian species have meanwhile been analyzed (Shear 2015; Bodner et al. 2016) and, apart from quinones, an increasing number of non-quinonic compounds has been reported from the secretions of several species (Schildknecht and Weis 1961; Röper and Heyns 1977; Vujisic et al. 2014; Shear 2015; Bodner et al. 2016). Particularly for representatives of the order Julida several aberrant defensive compounds have been described, including phenolics, straight-chain alcohols and aldehydes, and a series of long-chain esters (Wheeler et al. 1964; Wheaterston et al. 1971; Huth 2000; Vujisic et al. 2014; Bodner and Raspotnig 2012; Shimizu et al. 2012). Recently, Makarov et al. (2017) investigated the defensive secretions of several species of the so-called “Typhloiulini” (Julida, Julidae) including a recently described endogean species from Bulgaria, *Typhloiulus orpheus* Vagalinski, Stoev & Enghoff 2015. The secretion chemistry of *T. orpheus* was not completely elucidated, but shown to be predominated by one single unusual non-quinonic compound. We here provide detailed data on the unique defensive chemistry of this species.

## Materials and methods

### Collection of specimens

Adult individuals (two males, six females) of *Typhloiulus orpheus* were collected in Bulgaria, Western Rhodopi Mts., v. Trigrad, near Dyavolskoto garlo Cave, on limestone scree with sparse European spruce trees, 15–20 cm below the surface, at 41°36′54.51″N, 24°22′48.94″E (collection dates: May 27th 2014, Sept 2nd 2014, April 15th 2017, leg. B. Vagalinski). Vouchers are deposited at the National Museum of Natural History Sofia, Bulgaria.

### Extraction and analysis of defensive secretion

Defensive secretions were obtained by whole body extraction of single individuals in 200 μl of hexane for about 15 min. This method already proved to be suitable to gain secretions from small-seized juliformians (e.g., Makarov et al. 2017). Aliquots of extracts (1.5 µL) were analyzed by gas chromatography–mass spectrometry (GC–MS), using a Trace gas chromatograph coupled to a DSQ I mass spectrometer (MS), both from Thermo (Vienna, Austria). GC and MS conditions were the same as previously described in Bodner and Raspotnig (2012). Gas chromatographic retention indices (RI) of extract components were calculated using an alkane standard mixture (Van den Dool and Kratz 1963).

### Reference compounds

For comparison of GC–MS data, authentic 2-methyl-1,4-benzoquinone and methyl-*N*-methylanthranilate were purchased from Sigma (Vienna, Austria). For 2-ethyl-1,4-benzoquinone we used the defensive secretion of *Typhloiulus bureschi* and *T. georgievi* from which this compound had already been identified (Makarov et al. 2017).

## Results and discussion

**Fig. 1:**
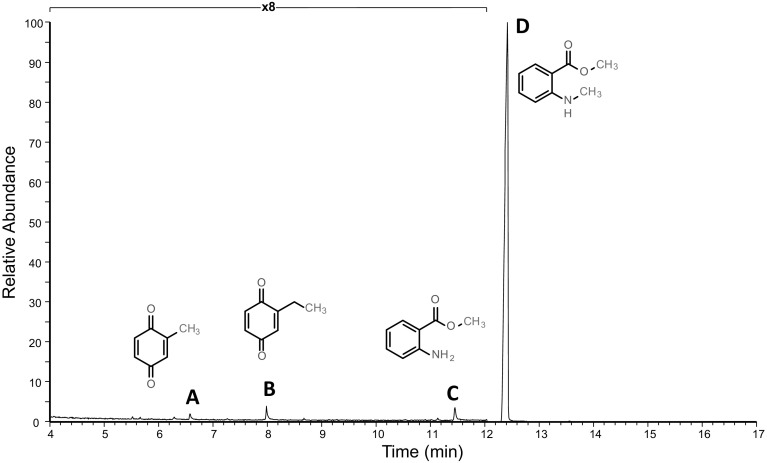
Characteristic chromatographic profile of the defensive secretion of *Typhloiulus orpheus*. Compound A (2-methyl-1,4-benzoquinone), compound B (2-ethyl-1,4-benzoquinone), compound C (methyl anthranilate), compound D (methyl *N*-methylanthranilate). Due to the minor amounts of compounds A–C, the first part of the chromatogram is displayed with magnification (×8)

The secretion of *T. orpheus* exhibited a stable pattern of four compounds (Fig. 1, compounds A–D). Analytical data to the identification of all four compounds are given in Table 1. Compounds A and B appeared to be substituted benzoquinones, showing abundant molecular ions at *m/z* 122 and *m/z* 138, respectively. These compounds were already familiar to us from previous studies (Bodner et al. 2016; Makarov et al. 2017) and were identified as 2-methyl-1,4-benzoquinone (toluquinone) and 2-ethyl-1,4-benzoquinone. Compounds C (M^+^ at *m/z* 151; RI = 1347) and D (M^+^ at *m/z* 165; RI = 1422) were indicated to contain nitrogen and were identified as an ester of *N*-methylanthranilic acid, methyl *N*-methylanthranilate (MNMA) and methyl anthranilate, respectively (Table 1). The major compound was MNMA, comprising about 99% of the secretion. Male and female profiles showed no differences.

**Table 1:** Gas chromatographic and mass spectral data to the secretion of *Typhloiulus orpheus*

Peak no.	Retention index RI measured (authentic reference^a^)	Mass spectrometric fragmentation *m/z* (rel. intensity)	Identified as	Secretion profile^c^
A	1014 (1014)	122 (M^+^, 100), 94 (52), 82 (35), 68 (19), 66 (26), 54 (40)	2-methyl-1,4-benzoquinone	0.2 ± 0.2
B	1106 (1107)	136 (M^+^, 90), 108 (100), 107 (28), 82 (36), 79 (72), 65 (7), 54 (51)	2-ethyl-1-4-benzoquinone	0.3 ± 0.2
C	1349 (1347^b^)	151 (M^+^, 84), 120 (23), 119 (100), 92 (43), 91 (4), 65 (15)	Methyl anthranilate	0.1 ± 0.1
D	1422 (1417)	165 (M^+^, 100), 134 (18), 133 (26), 132 (41), 116 (5), 106 (10), 105 (57), 104 (58), 91 (6), 78 (15), 77 (28)	Methyl *N*-methylanthranilate	99.4 ± 0.3

^a^Authentic standards (see “Materials and methods”) were used for index calculation, except for methyl anthranilate

^b^RI as reported by Bertrand et al. (2006)

^c^Secretion profiles are given in % of peak area of compounds relative to the total area of all secretion compounds

The “Typhloiulini”, a paraphyletic assemblage of cave- and non-cave dwelling julids, are already known for their aberrantly composed defensive secretions. Some typhloiuline representatives produce ethyl-benzoquinones (EBs) in addition to the more common methylated benzoquinones (Makarov et al. 2017). Though *Typhloiulus orpheus* is a representative of this EB-lineage of typhloiulines (2-ethyl-benzoquinone is present as a minor compound), it mainly produces MNMA, a novel compound for diplopod defensive chemistry. Esters of anthranilic acid, mainly methyl anthranilate, are well-known floral attractants for several insects such as hymenopterans, coleopterans, dipterans, and thrips (e.g., Murai et al. 2000; Larsson et al. 2003; James 2005). Methyl anthranilate has also been identified in different pheromonal roles from a number glands in ants, such as from mandibular, Dufour, and postpygidial glands, respectively (e.g., Duffield et al. 1980; Lloyd et al. 1984, 1989; Oldham et al. 1994). By contrast, MNMA—to our knowledge—has not been described from any exocrine gland of arthropods yet. Regarding chemical defense, anthranilate derivatives were described to represent effective repellents against insects such as mosquitos (e.g., Islam et al. 2017) and birds (Glahn et al. 1989; Clark et al. 2000; Müller-Schwarze 2009). For starlings, MNMA is highly aversive, leading to irritations of nerves associated with the olfactory system (Mason et al. 1989). Interestingly, starlings are known millipede predators, and their diet may even largely rely on certain julid species (e.g., Cloudsley-Thompson 1958). However, it has not yet been reported whether birds actively prey on *T. orpheus*.

Mechanisms triggering modifications of exocrine secretions are poorly known, and it is not axiomatic that secretions generally evolve via slow and small changes. Factors responsible for modifications in the chemical repertoire of exocrine secretions, such as the shift from widely homogenous benzoquinonic secretions (as common in juliform diplopods) to a different chemistry (such as MNMA in *T. orpheus*), may be due to changes in the ecological environment, and predation pressure must be considered to play the most important role. Rapid saltational shifts in the evolution of secretion chemistry may indeed occur and are regarded responsible for the sometimes completely distinct pheromones in closely related taxa (Symonds and Elgar 2004, 2008). Such pheromones may evolve in large steps by activation of ancestral genes (e.g., Roelofs et al. 2002), and the evolution of defensive secretions is likely to follow a similar pattern. In juliformians, shifts in defensive chemistry have been reported from several species: the julid diplopod *Allajulus dicentrus*, for instance, suddenly produces 2-alkenals as major secretion compounds, whereas the secretions of other *Allajulus* spp. contain the typical juliform benzoquinones but no other components (Bodner and Raspotnig 2012). *Rhinocricus padbergi*, a spirobolid, contains an unusual alkaloid, 3,3a,4,5-tetrahydro-1H-pyrrolo-[2,3-b]pyridine-2,6-dione, in its secretion (Arab et al. 2003). Saltational shifts may also be an explanation for the clearly distinct defensive chemistry in different taxonomic groups of a larger taxon; such chemically distinct taxonomic entities may be regarded the descendants of an early ancestor with already modified chemistry.

However, we consider that MNMA in *T. orpheus* did not arise via a completely new synthetic route but may indeed be linked to already existing biosynthetic pathways. Even though the biosynthetic mechanisms to defensive benzoquinones in millipedes are largely unknown, several authors consider benzoquinones in diplopods to originate from aromatic amino acids such as tryptophan (Duffey and Blum 1977; Blum 1981), and anthranilic acid is a known intermediate in tryptophan biosynthesis (Radwanski and Last 1995). Anthranilic acid is involved in tryptophan formation in bacteria, fungi, and plants, but also accrues by tryptophan degradation. Interestingly, anthranilic acid was reported to be a precursor of defensive compounds in another diplopod group as well: the secretions of glomeridans appear to exclusively contain unusual quinazolines, 1-methyl-2-ethyl-4(3H)-quinazoline (glomerin) and 1,2-dimethyl-4(3H)-quinazoline (homoglomerin) (Meinwald et al. 1966; Shear et al. 2011). There is evidence that anthranilic acid functions as precursor for these quinazolines as well, whereas homoglomerin can be further hydrolyzed via NaOH to MNMA (Schildknecht and Wenneis 1967; Blum 1981). In these terms, MNMA possibly emerges as a pivotal point in the biosynthesis of defensive compounds in representatives of least two classes of diplopods, linking together the complex biosynthetic machinery to diplopod defensive chemistry on a large scale.
